# Genome-Wide Signatures of Selection in *Colletotrichum kahawae* Reveal Candidate Genes Potentially Involved in Pathogenicity and Aggressiveness

**DOI:** 10.3389/fmicb.2019.01374

**Published:** 2019-06-19

**Authors:** Ana Vieira, Diogo Nuno Silva, Vitor Várzea, Octávio Salgueiro Paulo, Dora Batista

**Affiliations:** ^1^Centro de Investigação das Ferrugens do Cafeeiro, Instituto Superior de Agronomia, Universidade de Lisboa, Oeiras, Portugal; ^2^Computational Biology and Population Genomics Group, Centre for Ecology, Evolution and Environmental Changes, Faculdade de Ciências, Universidade de Lisboa, Lisbon, Portugal; ^3^Linking Landscape, Environment, Agriculture and Food, Instituto Superior de Agronomia, Universidade de Lisboa, Lisbon, Portugal

**Keywords:** genomics, coffee, host–pathogen interaction, coffee berry disease, host specialization, RAD-seq

## Abstract

Plants and their pathogens are engaged in continuous evolutionary battles, with pathogens evolving to circumvent plant defense mechanisms and plants responding through enhanced protection to prevent or mitigate damage induced by pathogen attack. Managed ecosystems are composed of genetically identical populations of crop plants with few changes from year to year. These environments are highly conducive to the emergence and dissemination of pathogens and they exert selective pressure for both qualitative virulence factors responsible for fungal pathogenicity, and quantitative traits linked to pathogen fitness, such as aggressiveness. In this study, we used a comparative genome-wide approach to investigate the genomic basis underlying the pathogenicity and aggressiveness of the fungal coffee pathogen *Colletotrichum kahawae* infecting green coffee berries. The pathogenicity was investigated by comparing genomic variation between *C. kahawae* and its non-pathogenic sibling species, while the aggressiveness was studied by a genome-wide association approach with groups of isolates with different phenotypic profiles. High genetic differentiation was observed between *C. kahawae* and the most closely related species with 5,560 diagnostic SNPs identified, in which a significant enrichment of non-synonymous mutations was detected. Functional annotation of these non-synonymous mutations revealed a significant enrichment mainly in two gene ontology categories, “oxidation–reduction process” and “integral component of membrane.” Finally, the annotation of several genes potentially under-selection revealed that *C. kahawae’s* pathogenicity may be a complex biological process, in which important biological functions, such as, detoxification and transport, regulation of host and pathogen gene expression, and signaling are involved. On the other hand, the genome-wide association analyses for aggressiveness were able to identify 10 SNPs and 15 SNPs of small effect in single and multi-association analysis, respectively, from which 7 were common, giving in total 18 SNPs potentially associated. The annotation of these genomic regions allowed the identification of four candidate genes encoding F-box domain-containing, nitrosoguanidine resistance, Fungal specific transcription factor domain-containing and C6 transcription factor that could be associated with aggressiveness. This study shed light, for the first time, on the genetic mechanisms of *C. kahawae* host specialization.

## Introduction

Plant diseases have become one of the most challenging threats to modern agriculture, not only for their huge economic impact caused by severe production losses, but also due to a global food security problem. Fungi are among the most devastating plant pathogens given their ability to overcome plant defenses and exploit the host’s resources for their own reproduction and dispersion (Möller and Stukenbrock, 2017). In fact, plants and their pathogens are involved in a continuous battle, with pathogens evolving to suppress plant defenses and plants responding through enhanced protection mechanisms to reduce or suppress pathogen damage, leading to a co-evolutionary dynamics that shapes the genomic landscape of both plants and pathogens (Zhan et al., 2014; Möller and Stukenbrock, 2017). In natural systems, this co-evolution is tempered by host and environmental heterogeneity as well as pathogen trade-offs between pathogenicity and several life style traits (Zhan et al., 2014, 2015). By contrast, in managed ecosystems, crops evolve through artificial selection, in which agriculturally desired traits are favored and the genetic heterogeneity of the host is severely reduced (Möller and Stukenbrock, 2017). In such homogeneous environments, the pathogen has a selective advantage, and newly pathogenic strains can quickly increase in frequency and spread across the fields (Zhan et al., 2014). The genetic homogeneity of these environments also means that pathogens spend more time in a single selective environment when compared to the wild system. Therefore, it is likely that the host exerts a selective pressure for quantitative traits linked to pathogen fitness, such as aggressiveness, as they do for qualitative virulence factors responsible for fungal pathogenicity (Elad and Pertot, 2014). Currently, the majority of plant pathogen studies are focused on the ability of the pathogen to infect the host (pathogenicity), and only few studies have focused their attention on the quantitative aspects of host–pathogen interactions (aggressiveness). However, it has been argued that it is the combination of these two approaches that will guide the formulation of sustainable disease management strategies that can minimize disease epidemics while simultaneously reduce pressure on pathogens to evolve and increase in pathogenicity and aggressiveness (Pariaud et al., 2009; Zhan et al., 2014, 2015). From an evolutionary perspective, it is well-known that the host is the strongest driver of pathogen evolution, as a successful infection is required for pathogen reproduction and dispersal. In this sense, genes related to pathogenicity are expected to be under strong selective pressure, and consequently, genomic signatures of selection can be used to identify candidate genes involved in host–pathogen interactions (Möller and Stukenbrock, 2017). However, the potential of pathogens to evolve in response to host selective pressures can also be constrained by trade-offs in quantitative traits, namely the rate of infection progression. In fact, the existence of phenotypic variation in aggressiveness is a key factor necessary for pathogen adaptation (Delmas et al., 2016). Hence, aggressiveness can be assessed by evaluating multiple phenotypic quantitative traits of the pathogen directly linked to its fitness. These traits are likely to be also under selection, resulting in differential adaptive patterns according to the environment (Pariaud et al., 2009).

Nowadays, thanks to the development of high-throughput sequencing (HTS) a new era in plant pathology has emerged, making possible to unveil the genetic mechanisms underlying the pathogenicity and aggressiveness of pathogens (Byers et al., 2016; Grünwald et al., 2016). Genome scans for detecting genomic regions under positive selection can be used to identify genes involved in adaptation, both within and between closely related species, while genome-wide association studies GWAS can identify genomic regions associated with a particular phenotype (Byers et al., 2016; Grünwald et al., 2016). Thus, a precise and reproducible measure of the relevant phenotype is the major limitation of GWAS (Talas et al., 2016). Both these approaches have been used in fungi to investigate host adaption (Connelly and Akey, 2012; Dalman et al., 2013; Palma-Guerrero et al., 2013; Gao et al., 2016; Talas et al., 2016), but their application is still in its infancy compared to model plant and animal systems. Moreover, the genes identified as putatively under selection or associated with a phenotype are only candidates that require further experimental testing to determine how they affect the phenotype (Grünwald et al., 2016).

*Colletotrichum kahawae* Waller & Bridge is a highly aggressive and specialized fungal pathogen, causing Coffee Berry Disease (CBD) in Arabica coffee in Africa. This pathogen emerged within the *C. gloeosporioides* complex, as a specialist pathogen with the ability to infect green coffee berries, an ecological niche previously unoccupied by other fungi (Silva et al., 2012). CBD can lead to severe production losses that reach up to 80% in extremely wet years, if no control measures are applied (Silva et al., 2006; Loureiro et al., 2012; Kebati et al., 2016; Alemu et al., 2017), and, for that reason, *C. kahawae* was ranked as a quarantine pathogen and considered as a biological weapon (Kebati et al., 2016; Batista et al., 2017). Consequently, the pathogen’s potential dispersal to other Arabica coffee cultivation regions is greatly feared, particularly to those at higher altitudes in Latin America and Asia. So far, no absolute effective control measure has been developed but some *Coffea* spp. genotypes show high levels of resistance (Várzea et al., 2002). *C. kahawae* has also been described as a pathogen with a low genetic variability, clearly structured into three clonal and completely differentiated populations (Angolan, Cameroonian, and East African) (Silva et al., 2012; Vieira et al., 2018), and two clonal lineages within the Angolan population (Vieira et al., 2019). Furthermore, significant differences in aggressiveness of isolates were consistently observed, regardless of their geographic origin (Bridge et al., 2008; Loureiro et al., 2011; Pires et al., 2016). Recently, Vieira et al. (2019) performed a comprehensive analysis and characterization of *C. kahawae* aggressiveness trait, establishing three main aggressiveness classes (high, moderate, and low). By providing consistent phenotypic data on aggressiveness, this study brought the opportunity to perform a GWAS for this trait in this pathogen.

Up to now, and in contrast with other *Colletotrichum* species, little is still known about the adaptive genetic variation of *C. kahawae* and no reports have been made on candidate genes underlying its pathogenicity and/or aggressiveness. Therefore, the current work aims to: (i) understand the genomic basis underlying the pathogenic behavior of *C. kahawae* on green coffee berries using a genomic comparative analysis with closely related non-pathogenic fungi, and (ii) identify the genomic regions potentially associated with aggressiveness through a GWAS. These results will contribute to better understand the genomic basis underlying these two complex processes, which may allow the establishment of more evidence-based and effective control measures in the future.

## Materials and Methods

### Sampling, DNA Isolation, and RAD – Sequencing

In this work, 30 *C. kahawae* isolates (CIFC/ISA/ULisboa collection) representative of the three genetic groups described by Silva et al. (2012), and covering almost all regions where the disease exists (10 African countries) were used, as well as 10 isolates from non-pathogenic sibling species collected from different hosts and several countries across the world (Supplementary Table S1). According to Weir et al. (2012), these latter isolates belong to three different species [*G. cingulata* “f.sp. camelliae,” *C. aotearoa* and *C. kahawae* subsp. *ciggaro* (*Cc*)]. However, in this study, the two *C. kahawae* subspecies *sensu* Weir et al. (2012) are accepted as cryptic species as suggested by Batista et al. (2017) and described accordingly. Culturing and DNA extraction from fungal isolates were performed as previously described by Silva et al. (2012), with slight modifications. Briefly, isolates were grown in liquid media containing 3% malt extract and 0.5% peptone, under a photo-period of 12 h at 22°C. DNA was extracted from freeze dried mycelia with the Sigma Plant/Fungi DNA isolation kit (Sigma-Aldrich, Darmstadt, Germany), according to the manufacturer’s instructions. Genomic DNA quality was evaluated by agarose gel and quantified using a Thermo Scientific (Waltham, MA, United States) Nanodrop ND-1000 spectrophotometer. Three micrograms of high-quality genomic DNA per sample were sent to Floragenex, Inc. (Portland, OR, United States) for RAD library preparation and sequencing. Libraries with sample-specific barcode sequences [8 nucleotide (nt)] were produced from DNA digested with PstI. RAD-seq pools were 100 bp single-end-sequenced in a lane of an Illumina HiSeq 2000 machine. The sequence data was deposited in the European Nucleotide Archive under Accession Nos. PRJEB26929 and PRJEB28813.

### RADseq Quality Filtering and SNP Calling

Sequence reads were de-multiplexed and quality filtered with the process_radtags program from the package Stacks v1.20 (Catchen et al., 2013). Reads with uncalled bases or distance to barcodes higher than 1 were removed. Base calls with a Phred score under 20 were converted to Ns and reads containing more than 4 Ns were discarded. Barcodes and Illumina adapters were excluded from each read and length was truncated to 85 bp (-t 85). Additional filtering, and *de novo* assembly within and between individuals to identify loci was performed using the program PyRAD v3.0.5 (Eaton, 2014). This software was chosen due to its ability to handle indels when clustering sequence reads into orthologous loci. In this study, several clustering parameters were tested in order to minimize the number of missing data and maximize the number of phylogenetic informative sites (Supplementary Table S2). The sequence variants [single nucleotide polymorphisms (SNPs)] were then exported into a variant call format (VCF) and the “stacks” information exported as a loci file. Handling and exploration of alignment data matrices were performed using TriFusion v1.0.0 software^1^.

### Phylogenetic Analysis

To assess phylogenetic relationships among the isolates we used a single concatenated alignment that includes loci with SNPs represented in more than 80% of the isolates and a minor allele frequency (MAF) above 5% (*total_dataset*). Concatenation and conversion of the alignment matrices to the appropriate formats was performed with TriFusion. A maximum likelihood analysis was conducted with RAxML v. 8. 2 (Stamatakis, 2014) on the CIPRES Portal (Miller et al., 2010), using the general time-reversible (GTR) model of nucleotide substitution with the CAT distributed rate heterogeneity. Non-parametric bootstrapping was performed with the fast bootstrap algorithm of RAxML with 1000 replicates using the GTRCAT substitution model. Bayesian inference was performed using MrBayes v3.2.6 (Ronquist et al., 2012) with the GTR +Γ model of sequence evolution. The best-fitting model was determined according to the Akaike information criterion (Posada and Buckley, 2004). Posterior probabilities were generated from 1 × 10^7^ generations, sampling at every 1000th iteration, and the analysis was replicated three times with one cold and three incrementally heated Metropolis-coupled Monte Carlo Markov chains, starting from random trees. The achievement of the stationary phase and mixing was checked for all parameters using Tracer V1.4, and 1 × 10^6^ generations (corresponding to 10% of the total of generations) were discarded as burn-in. Trees from different runs were combined using Logcombiner and summarized in a majority rule 50% consensus tree. All trees were visualized in FigTree^2^ and further edited in Inkscape^3^. Regardless of the dataset under study (datasets generated with different PyRAD parameters), a similar phylogenetic tree was reconstructed.

### Detection of Genomic Signatures of Positive Selection Related to the Pathogenicity of *C. kahawa*e

**FIGURE 1:**
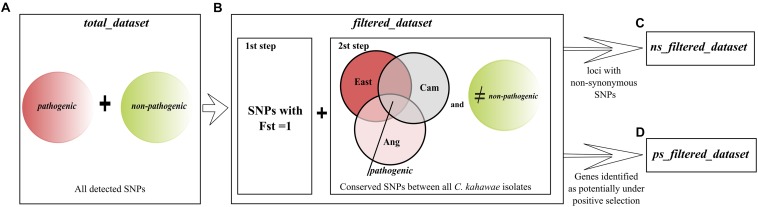
Schematic representation of the datasets used for the analyses conducted in this study. **(A)** *total_dataset* comprising all the detected SNPs; **(B)** *filtered_dataset* comprising the diagnostic SNPs between pathogenic and non-pathogenic groups. The three *Colletotrichum kahawae* populations were named as Ang (Angolan), Cam (Cameroonian), and East (East African). **(C)** *ns_filtered_dataset* comprising all the loci with non-synonymous SNPs within the diagnostic SNPs; **(D)** *ps_filtered_dataset* comprising all the genes potentially under positive selection.

In this study, pathogenic (*C. kahawae*) and non-pathogenic fungi (*G. cingulata* “f.sp. *camelliae*,” *C. aotearoa* and *Cc*) to Arabica coffee were analyzed in order to better understand the pathogenicity of *C. kahawae*. The initial dataset named as *total_dataset* comprise all the genetic variation observed within the species (Figure 1A). In addition, a second dataset named *filtered_dataset* was constructed using the diagnostic SNPs, i.e., the SNPs completely differentiated between pathogenic and non-pathogenic groups, which were selected with the following sequential filters: (i) by calculating the distribution of SNPs Fst values using VCFTOOLS v0.1.14 (Danecek et al., 2011) and Arlequin v3.5.2 (Excoffier and Lischer, 2010) and choosing the SNPs with a Fst value equal to 1; (ii) by choosing the SNPs that were conserved across all *C. kahawae* isolates and completely differentiated from at least one of the non-pathogenic fungi (Figure 1B). Both datasets, *filtered_dataset* and *total_datase*t, were mapped against the genome of the most closely related species within the *Colletotrichum* genus [*C. fruticola* (previously mis-identified) as *C. gloeosporioides* Nara gc5 (Baroncelli et al., 2016), accession_number (GCA_000319635.1) and reference (SAMN02981487)]. A copy of the assembled scaffolds was obtained from the Ensembl Genome Browser^4^. All loci were then aligned to the reference genome using Bowtie 2.2.1.0 (Langmead and Salzberg, 2012) with the “–very-sensitive-local default” setting. Alignments were sorted with SAMTools 0.1.19 (Li et al., 2009) and the loci that aligned to more than one location were removed from the analysis. The SNPs location, annotation, and classification of type of mutation were assessed with a custom-made python script available on https://github.com/yanavieira/Mapping_SNPs_Genome.git. At this point an additional filtration step was incorporated and the non-synonymous mutations identified in the *filtered_dataset* were used to create a new dataset named *ns_filtered_dataset* (Figure 1C). The consensus of the RADseq loci of the three datasets (*total_dataset, filtered_dataset*, and *ns_filtered_dataset*) was functionally annotated. The categorization was made through a similarity BLASTx search using Blast2GO (Gotz et al., 2008), against the NCBI non-redundant database with a minimum expectation value of 10^–3^, and the remaining functional annotation was carried out using the default parameters. The Gene Ontology (GO) terms were assigned to the 2nd level of the biological process, molecular protein and cellular component categories. A GO enrichment analysis was performed to determine if any GO term was over or under represented in the *filtered_dataset* and *ns_filtered_dataset* when compared to the *total_dataset*. Statistically significant enrichment was tested against a reference of all genes analyzed using the Fisher’s exact test and a significance of FDR < 0.05.

The dN/dS ratio was measured for the genes identified in the *filtered_dataset*, and those having a ratio higher than 1 were considered as candidate genes under positive selection and assembled as the *ps_filtered_dataset* (Figure 1D). The annotation of these genes was further improved by searching with BLASTx the *C. kahawae* Rad loci (E-value ≤ 1e-1) and the orthologous *C. gloeosporioides* genes (E-value ≤ 1e-9) against the pathogen–host interaction reference database (PHI-base) v.4.2 (Urban et al., 2017).

### Genome Wide Association Analyses for *C. kahawae* Aggressiveness

The dataset used to perform the GWAS (*gwa_dataset*) was filtered in three steps to remove: (i) all non-pathogenic fungi to green coffee berries; (ii) all SNPs that contributed to the genetic structuring within *C. kahawae*, since the power of GWAS can be significantly reduced by the inclusion of related individuals and population substructure (Connelly and Akey, 2012); (iii) four isolates of *C. kahawae* that were not phenotypically classified by Vieira et al. (2018).

**FIGURE 2:**
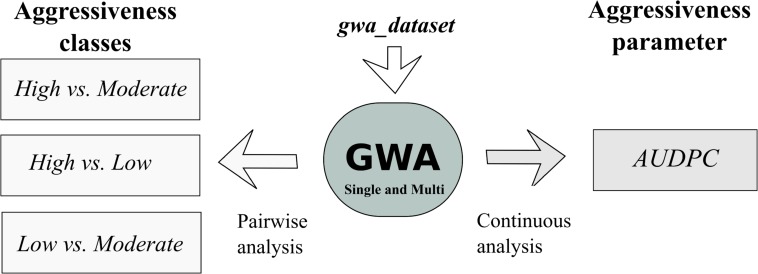
Schematic representation of the dataset and GWA analyses conducted in this study. The pairwise analysis was performed taking into account the aggressiveness classes (*High*, *Moderate*, *Low*) previously described by Vieira et al. (2018), and the continuous analysis was performed with the *AUDPC* values obtained by Vieira et al. (2018).

The Bayesian variable selection regression (BVSR) model proposed by Guan and Stephens (2011) and implement in piMass v 0.9 has the ability to perform single and multi-SNP association analyses using not only binary phenotypes but also continuous response variables. In this work, we applied BVSR to perform a single and multi-SNP correlation analysis between SNP alleles and the aggressiveness phenotype using a pairwise comparative analysis between the three aggressiveness classes established by Vieira et al. (2018) (*High*, *Moderate*, and *Low*), and a continuous analysis with the Area Under the Disease Progress Curve (*AUDPC*) parameter recorded for each isolate by Vieira et al. (2018). A schematic representation of all the analyses performed and the datasets used is illustrated in Figure 2.

The single-SNP association analysis allowed the detection of the associated SNPs even in the absence of interactions between them (Guan and Stephens, 2011). In this analysis, the SNPs with an empirical quantile for Bayes factor (BF) above 97.5% (BF0.975 SNPs) were considered as strongly associated with isolates’ aggressiveness. By contrast, the multi-SNP association analysis, uses the phenotype as the response variable and the genetic variants (SNPs) as covariates to evaluate SNPs that may be associated with a phenotype (Guan and Stephens, 2011). SNPs statistically associated with phenotypic variation were identified by the posterior distribution of γ, or the posterior inclusion probability (PIP). In our association analyses, markers with a PIP greater than 97.5% empirical quartile (PIP 0.975 SNPs) were considered as highly associated with an aggressiveness class. For all 0.975 SNPs the respective PIP and the estimates of their phenotypic effect (β) are reported. A positive β in the pairwise X-Y aggressiveness class analysis means that the frequency of the MAF is higher in the Y aggressiveness class and a negative β means that MAF is higher in the X aggressiveness class. Thus, to investigate the phenotypic effect size of each PIP0.975 SNP, the |β| was considered. Additional parameters contained in the model were estimated from the data: proportion of variance explained by the SNPs (PVE), the number of SNPs in the regression model (nSNPs) and the average of phenotypic effect of the SNP contained in the model (σSNP). For all pairwise and continuous analyses, we obtained 4 million Markov Chain Monte Carlo samples from the joint posterior probability distribution of model parameters (recording values every 400 iterations), and discarded the first 100,000 samples as burn-in. Imputation of missing genotypes was performed in BIMBAM v1.0 (Servin and Stephens, 2007), in which the state of a non-genotyped marker is inferred from the haplotype of the other individuals. The loci where the SNPs potentially associated with the aggressiveness trait are located, regardless the type of GWAS analysis, were functionally annotated as previously described in Section “Detection of Genomic Signatures of Positive Selection Related to the Pathogenicity of C. kahawae,” including the search on PHI-base for the SNPs located in coding regions.

## Results

### RAD Tag Generation and *de novo* Assembly

Illumina RAD-seq of 30 *C. kahawae* isolates, collected from almost all coffee regions where CBD occurs, and 10 isolates from several closely related species of the *C. gloeosporioides* complex, generated an average of 3.76 × 10^6^ reads per sample, amounting to a total of 150.41 × 10^6^ of 85 bp single-end reads after barcode trimming. The individual read number ranged between 1.46 × 10^6^ and 6.14 × 10^6^, after an initial quality filter to remove the low-quality reads, in which an average of 5.83 × 10^5^ reads were discarded. Ten *de novo* assemblies were performed, and the results are summarized in Supplementary Table S2. The best *de novo* assembly, i.e., the one that minimizes the number of missing data and maximizes the number of phylogenetically informative sites, was obtained with the following parameters: *minimum depth of coverage of* 10, *maximum number of low quality of* 4, *clustering threshold of* 0.90, *minimal taxon coverage of* 5, and *maximum shared* heterozygosity *of* 3. Additional filtering steps, including the removal of SNPs with less than 80% of the taxa represented and a MAF lower than 5%, yielded a final matrix of 83,528 SNPs across 28726 loci and 40 isolates, referred as *total_dataset*.

### Phylogenetic Analysis

**FIGURE 3:**
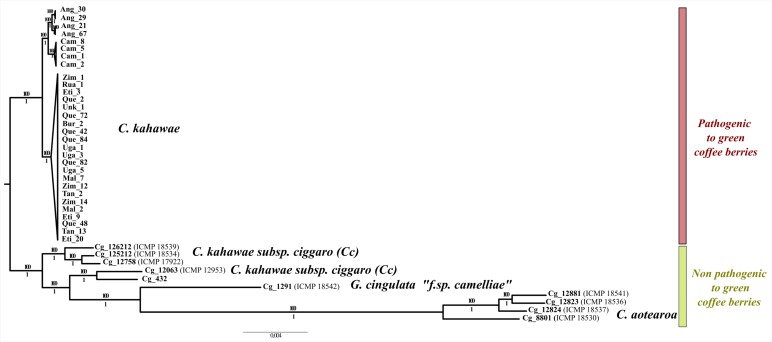
Maximum likelihood phylogenetic tree illustrating the evolutionary relationships among pathogenic and non-pathogenic fungi to green coffee berries. Bootstrap and posterior probability values are provided above and below the branches, respectively.

The phylogenetic analysis of the *total_dataset* produced a completely resolved evolutionary tree for the *C. gloeosporioides* complex species under study (Figure 3). Overall, a clear genetic differentiation was observed between the pathogenic and non-pathogenic species. The branches were well-supported in both analyses (Maximum Likelihood and Bayesian analyses) with all species being monophyletic, except for *Cc* that seems to be paraphyletic. In fact, two isolates (ICMP_12953 and Cg_432) are more differentiated from the remaining *Cc* isolates and may even belong to a different species. The most differentiated species of the *C. gloeosporioides* complex under study was *C. aotearoa*. Finally, a geographical structuring within *C. kahawae*, like the one previously described by Vieira et al. (2019) was observed, in which three well-supported populations (Angolan, Cameroonian, and East African) and two clonal lineages within Angolan population are evident.

### Genomic Regions Underlying the Pathogenicity of *C. kahawa*e

In this study, the isolates were sorted into two groups, pathogenic and non-pathogenic, according to their ability to infect green coffee berries. The pathogenic group has all *C. kahawae* isolates (comprising 3,297 SNPs), while the non-pathogenic group has isolates from the three closely related species (*G. cingulata* “f.sp. *Camelians*,” *C. aotearoa* and *Cc*) with a total of 71,503 SNPs. The genetic variability between the two groups comprises 83,528 SNPs, which as previously referred, constitutes the *total_dataset* (Figure 1A). From this dataset, diagnostic SNPs were chosen based on two sequential filtering steps (Figure 1B). The first filtering led to the identification of 7,773 SNPs located in 5,974 loci that are completely differentiated between the two groups (Fst = 1), while the second step reduced the data matrix to a final group of 5,560 diagnostic SNPs located in 4,619 loci across 40 isolates, referred as *filtered_dataset*.

**FIGURE 4:**
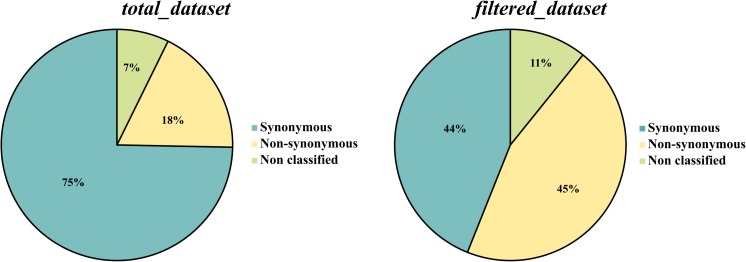
Comparative analysis of the number of synonymous and non-synonymous SNPs in *total_dataset* and *filtered_dataset.*

Both datasets, *total_dataset* and *filtered_dataset*, were mapped against the genome of the most closely related species within the genus *Colletotrichum*, *C. fruticola* (Nara gc5). Only 28% (23,613 SNPs) of the *total_dataset* and 34% (1,869 SNPs) of the *filtered_dataset* were successfully mapped. This analysis revealed that, in the *total_dataset*, 47% (11,162 SNPs) are located in non-coding regions, 53% (12,444 SNPs) are located in genes and 7 SNPs in pseudo genes, while in the *filtered_dataset*, 55% (1,019 SNPs) are located in non-coding regions, 45% (847 SNPs) are located in genes and 3 SNPs are located in pseudo genes. Regarding the number of synonymous and non-synonymous mutations, a significant increase on the number of non-synonymous mutations was found in the *filtered_dataset* (45%) when compared to the *total_dataset* (18%) (Figure 4). The type of mutation in *filtered_dataset* was further used to select the diagnostic SNPs that lead to a non-synonymous mutation (*ns_filtered_dataset*-Figure 1C) and to screen the genes under positive selection (*ps_filtered_dataset-*Figure 1D). The *ns_filtered_dataset* contains 348 non-synonymous diagnostic SNPs located in 336 loci, while the *ps_filtered_dataset* contains 258 genes under positive selection (dN/dS ratio > 1), from which 26 had more than 1 non-synonymous mutation.

**FIGURE 5:**
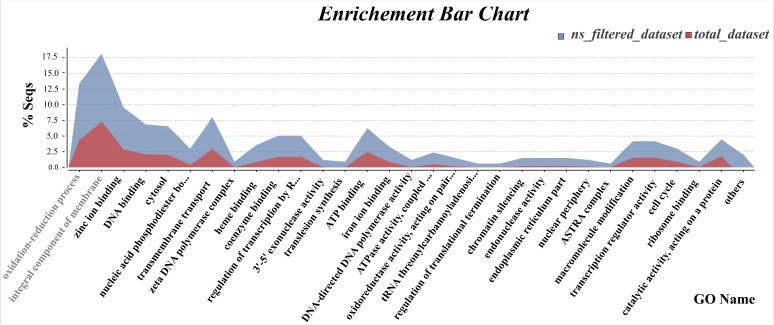
Enrichment of gene functional categories among *total_dataset* and *ns_filtered_dataset*. Curve chart comparing the proportion of genes per GO term between *ns_filtered_dataset* and *total_dataset* with a statistically significance of FDR < 0.05, according to the Fisher’s exact test. The two most distinct GO term categories are evidenced in light gray.

Functional annotation of RADseq loci for the three datasets (*total_dataset*, *filtered_dataset*, and *ns_filtered_dataset*) was performed, in which 34, 29, and 87% respectively, matched to known genes in the NCBI nr database. GO terms were assigned to 25% of the *total_dataset*, 16% of the *filtered_dataset* and 57% of the *ns_filtered_dataset*, and analyzed at the 2nd level of functional annotation for biological process, molecular function and cellular component categories. Only small differences were observed between these three datasets (Supplementary Figure S1 and Supplementary Table S3). For the biological processes’ category, genes involved in “metabolic” and “cellular process” were highly represented in all datasets, while genes involved in “cellular component organization” were only present in the *total_dataset*. For the molecular function category, “binding” and “catalytic activity” is the most represented GO term, being the “transporter activity” specific to both *filtered_dataset* and *ns_filtered_dataset*. For the cellular component category, the mostly represented functional classes in all datasets were “cell,” “cell part,” “membrane,” and “membrane part.” Additionally, fisher’s exact test between *ns_filtered_dataset* and *total_dataset* revealed a significant enrichment of genes annotated into several GO terms, particularly for the functional classes “oxidation–reduction process” and “integral component of membrane” (Figure 5), while no significant differences were observed between the *filtered_dataset* and the *total_dataset*. Several genes identified as members of these two functional classes were over-represented, such as the gene encoding cytochrome P450 in both classes, FAD dependent oxidoreductase and nitric oxide synthase in the “oxidation–reduction process” class, and several transports namely, MFS transporter, ABC-2 type transporter and ammonium/hexose transporters in the “integral component of membrane” class (Supplementary Table S4).

The potential virulence role of the 258 genes found under positive selection was searched on the pathogen–host interaction database (PHI-base), using two distinct approaches: (i) blasting the complete gene retrieved from *C. fruticola* genome where the RAD loci mapped; and (ii) blasting the RAD loci of *C. kahawae* obtained during this study. A total of 77 *C. fruticola* genes had homology in the PHI-base, from which 40 gave also a match for *C. kahawae’s* RAD loci (Supplementary Table S5). From the total genes with a hit, 41 genes were reported to show a relevant role in fungal pathogenicity and virulence when a mutant phenotype was produced in other host–pathogen interactions (Supplementary Table S6). The majority of these genes belonged to the category of “reduced virulence” in the PHI-base, while others belonged to different categories, including “loss of pathogenicity” (chitin synthase, GTP-binding protein, ATP-binding cassette (ABC) transporter, alpha-mannosyltransferase *cmt1* and cytochrome p450) and “lethal” (ataxia telangiectasia mutated, C6 transcription factor, protein transport protein, ccr4-not transcription complex subunit) (Supplementary Table S6). Overall, the genes detected as being under positive selection, and consequently, with a putative role in the pathogenicity of *C. kahawae* are mainly involved in oxidation–reduction processes and transport, but signaling, binding and biosynthesis seems to have additional important roles in the infection process (Supplementary Table S5).

### Genome-Wide Association Study for the Phenotypic Trait of Aggressiveness

After filtering the data for the GWAS, 173 SNPs located in 141 loci across 26 isolates were identified. This dataset was used to test for associations of the population genetic variation with the phenotypic trait of aggressiveness. The efficiency of the filtering correction for the effect of population genetic structure can be confirmed in Supplementary Figure S2, showing that the selected SNPs were unable to recover the structuring pattern characteristic of *C. kahawae*.

The Single-SNP analysis, for all pairwise combinations (*High* vs. *Moderate*, *High* vs. *Low*, *Low* vs. *Moderate)* and continuous analysis (*AUDPC*), identified a total of 10 SNPs with BF0.975 (>97.5 quantile Bayes factor) associated with aggressiveness, corresponding to 6% of the analyzed markers. When a stricter threshold was applied (99% quantile), 6 BF0.99 SNPs (3.5%) showed the strongest association with aggressiveness (41944.81; 34174.84; 18945.9; 18945.8; 12430.32; 46939.8). The number of SNPs identified in *High* vs. *Low* class was always two regardless the threshold used, while for the remaining pairwise analyses (*High* vs. *Moderate* and *Low* vs. *Moderate*) and continuous analysis (*AUDPC*) the number of SNPs ranged from 5 to 2 when a more restricted threshold was applied (Table 1 and Supplementary Figure S3).

**TABLE 1:** SNPs associated with aggressiveness for each pairwise comparison (*High* vs. *Moderate*, *High* vs. *Low*, *Low* vs. *Moderate*) and for the continuous analyses (*AUDPC*) obtained through Single-SNP association tests using Bayesian regression approach.

	**Alternative**	**Reference**	**SNP**				**PHI-base**	**Phi-base**
**SNP_ID**	**allele**	**allele**	**location**	**BF0.975**	**|β|**	**Blast_hit**	**(*Cf* gene)**	**(*Ck* RADloci)**
*High* vs. *Low*								
41944.81 a	G	T	CoR	0.08	0.06	F-box domain-containing	No hits	No hits
34174.84 a,b	C	T	CoR	0.08	0.06	Nitrosoguanidine resistance	No hits	No hits
Mean_BF0.975					0.06			
Mean all SNPs					–0.02			
*High* vs. *Moderate*								
18945.5	A	G	NcR	0.16	0.07	Hypothetical protein	No hits	No hits
41944.81 a	G	T	CoR	0.16	0.07	F-box domain-containing	No hits	No hits
34174.84 a,b	C	T	CoR	0.28	0.08	Nitrosoguanidine resistance	No hits	No hits
35951.85	T	C	CoR	0.14	0.06	Fungal specific transcription factor domain-containing	FZC28	GzZC278
Mean_BF0.975					0.07			
Mean all SNPs					0			
*Low* vs. *Moderate*								
18945.9 a,b	C	T	NcR	0.18	0.09	Hypothetical protein	No hits	No hits
14003.77 b	T	G	NcR	0.14	0.08	—NA–	x	No hits
46939.81 a,b	T	A	X	0.21	0.09	—NA–	x	No hits
7756.83	C	A	X	0.18	0.09	—NA–	x	No hits
Mean_BF0.975					0.09			
Mean all SNPs					–0.02			
*AUDPC*								
18945.8 a,b	A	T	NcR	0.22	–1.86	Hypothetical protein	No hits	No hits
18945.6 b	C	T	NcR	0.2	–1.8	Hypothetical protein	No hits	No hits
12430.32 a,b	A	G	NcR	0.57	–2.49	—NA–	x	No hits
14003.77	T	G	NcR	0.19	–1.74	—NA–	x	No hits
34174.84	C	T	CoR	0.16	1.68	Nitrosoguanidine resistance	No hits	No hits
Mean_BF0.975					–1.24			
Mean all SNPs					0			

*^a^SNPs also selected with a BF 0.99; ^b^SNPs identify.*

**FIGURE 6:**
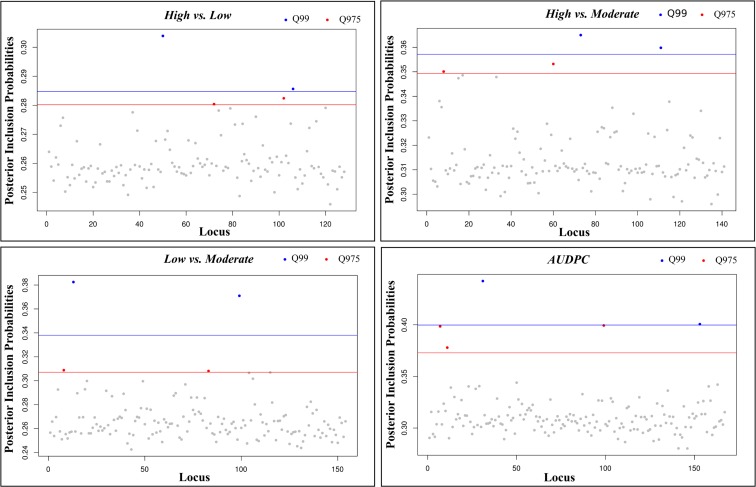
Posterior inclusion probabilities (PIPs) for each SNP in each pairwise comparison in multi-SNP association test. The horizontal blue lines correspond to the PIP 99% empirical quantile threshold and red lines to the 97.5% empirical quantile. Blue dots: SNPs with a PIP > 99% empirical quantile, Red dots: SNPs with a PIP > 97.5% empirical quantile, Light gray dots: SNPs with a PIP < 97.5% empirical quantile.

For the multi-SNP association analysis, estimates of the mean number of SNPs (nSNPs) underlying the aggressiveness variation ranged from 26.5 to 52.2 SNPs (Supplementary Table S7). When considering only models with the highest BFs [log10(BF) > BF0.99], the mean number of SNPs included in the model (nSNPs_BF) for each comparison decreased to values between 8.6 and 30.1, while the mean effect size of the SNPs (σSNP_BF) increased, ranging between 2.13 and 4.72 (Supplementary Table S7). The PIPs for the analyzed SNPs were similar among all analyses but slightly higher in the pairwise comparisons involving *High* vs. *Moderate* (PIP = 0.314) classes and the continuous analyses with the *AUDPC* values (PIP = 0.312) (Supplementary Table S7). In multi-association analysis, a subset of 5 SNPs, with the highest inclusion probabilities (PIP0.975), were detected for all pairwise combinations (*High* vs. *Moderate*, *High* vs. *Low*, *Low* vs. *Moderate*) and continuous analysis (*AUDPC*) (Figure 6). Estimates of the strength of association between genotype variation at individual SNPs and phenotypic variation (|β|) were always greater than 0.36, but changed according to the analyses (Table 2). Overall, we obtained SNPs with larger effect in the continuous analysis than in the remaining pairwise analyses. Three PIP0.975 SNPs were shared between at least two pairwise analyses (34174.84; 18945.7; 14003.77), and no common SNP were detected between the continuous and all the pairwise analyses. In total, 15 different SNPs revealed a multi-SNP association with aggressiveness, from which 7 were also significant in the single-SNP analyses (34174.84; 18945.9; 14003.77; 46939.81; 18945.8; 18945.6; 12430.32). Despite that, no causal SNP, i.e., able to explain the total phenotypic variation observed, was detected.

**TABLE 2:** SNPs associated with aggressiveness for each pairwise comparison (*High* vs. *Moderate*, *High* vs. *Low*, *Low* vs. *Moderate*) and for the continuous analyses (*AUDPC*) obtained through multi-SNP association tests using Bayesian regression approach.

	**Alternative**	**Reference**	**SNP**				**PHI-base**	**Phi-base**
**SNP_ID**	**allele**	**allele**	**location**	**PIP 0.975**	**|β|**	**Blast_hit**	**(*Cf* gene)**	**(*Ck* RADloci)**
*High* vs. *Low*								
18638.64 a	G	A	x	0.3	0.61	—NA–	x	No hits
41138.71	G	A	NcR	0.28	0.37	—NA–	x	No hits
34174.84 b	C	T	CoR	0.28	0.43	Nitrosoguanidine resistance	No hits	No hits
44503.84 a	A	G	CoR	0.29	0.45	C6 transcription factor	GzZC184	FZC55
*High* vs. *Moderate*								
18945.7	G	C	NcR	0.35	0.68	Hypothetical protein	No hits	No hits
17838.69	C	T	CoR	0.35	0.65	Hypothetical protein	SrbA	No hits
14003.77 a	T	G	NcR	0.37	0.79	—NA–	x	No hits
34174.84 a,b	C	T	CoR	0.36	0.66	Nitrosoguanidine resistance	No hits	No hits
*Low* vs. *Moderate*								
18945.7	G	C	NcR	0.31	0.57	Hypothetical protein	No hits	No hits
18945.9 a,b	C	T	NcR	0.38	0.92	Hypothetical protein	No hits	No hits
14003.77 b	T	G	NcR	0.31	0.49	—NA–	x	No hits
46939.81 a	T	A	x	0.37	0.87	—NA–	x	GIT3
*AUDPC*								
18945_6	T	A	NcR	0.56	0.71	Hypothetical protein	No hits	No hits
18945_8 b	A	T	NcR	–0.37	0.62	Hypothetical protein	No hits	No hits
12430_32 a,b	A	G	NcR	–0.49	0.89	—NA–	x	No hits
1691_81	G	A	CoR	0.51	0.79	—NA—	No hits	No hits
28376_85 a	T	C	NcR	–0.54	0.73	Hypothetical protein	x	No hits

*^a^SNPs also selected with a PIP 0.99; ^b^SNPs identified as potentially associated in single and multi-association analyses; CoR, coding region; NcR, non-coding region; x, no information.*

In total, 18 different SNPs putatively associated with aggressiveness were retrieved from both analyses. The loci containing those SNPs were BLASTed and functionally annotated, being described as genes located into “integral component of membrane” and “nucleus.” The search for the virulence role was performed in the PHI-base, revealing that the mutant phenotype for two transcription factors (FZC28 and GzZC184) was described as unable to affect pathogenicity in other host–pathogen interactions, while for other two genes (*GIT3*, *srbA*) the mutant phenotypes showed reduced virulence in *Candida albicans* and *Aspergillus fumigatus*, respectively (Supplementary Table S8). Finally, most of the SNPs putatively associated with aggressiveness in the single and multi-association analyses are in non-coding regions, with only six SNPs (34174.84; 44503.84; 17838.69; 1691.81; 41944.81; 35951.85) located in coding regions (Tables 1, 2). The annotation of these genomic regions allowed the identification of four candidate genes coding for F-box domain-containing, nitrosoguanidine resistance, Fungal specific transcription factor domain-containing and C6 transcription factor and several hypothetical proteins that could be associated with aggressiveness (Tables 1, 2).

## Discussion

### Phylogenetic Relationships and Host Specialization

One of the first and most striking findings of this work was the high genetic differentiation, at the genomic level, between pathogenic and non-pathogenic fungi to green coffee berries, reinforcing the idea that *C. kahawae* should be considered as a distinct species. The phylogenetic analysis, besides confirming the clear pattern of population structure proposed by Vieira et al. (2019), also revealed that *Cc* is the only paraphyletic group, and consequently, the possibility of this group harboring in fact more than one species cannot be discarded and should be further investigated. The non-pathogenic isolate most closely related with *C. kahawae* was Cg126212 (ICMP18539) instead of Cg_432 as previously referred by Silva et al. (2012), which reinforces the importance of using a large number of loci to capture a more accurate phylogenetic relationship. The remaining phylogenetic results corroborate the taxonomic classification proposed by Weir et al. (2012), and place *C. aotearoa* as the most distant species of the *C. gloeosporioides* complex under study.

### Footprints of Genomic Adaptation and Candidate Genes for Pathogenicity in *C. kahawa*e

In this study, we identified 5,560 diagnostic SNPs potentially involved in the pathogenicity of *C. kahawae* to green coffee berries. Although it is not probable that all these SNPs are related to this specific trait, the probability of finding the genetic variation involved in *C. kahawae‘s* pathogenicity is quite high. In fact, the enrichment in non-synonymous mutations found in this dataset (*filtered_dataset*) when compared to the *total_dataset* is quite promising, especially because this pattern was not observed for the total number of SNPs located in non-coding regions and/or coding regions. Functional annotation of these non-synonymous SNPs (*ns_filtered_dataset*), as well as of the diagnostic SNPs (*filtered_dataset*) and those of the *total_dataset*, revealed, for the 2nd level of the “biological process,” “molecular function,” and “cellular component” categories, only small differences between the datasets. In the “biological process” category, genes involved in “cellular component organization or biogenesis” are only present in the *total_dataset*, while for the remaining datasets a small enrichment in genes involved in “response to stimulus” was observed. In the “molecular protein” category, the genes involved in “transporter activity” seems to be over represented in both filtered datasets (*filtered_dataset* and *ns_filtered_dataset*), which could suggest that transporters have an important role in the pathogenicity of *C. kahawae*. Additionally, a significant enrichment of specific GO terms was detected in the *ns_filtered_dataset* when compared to the *total_dataset*, particularly the “oxidation–reduction process” and “integral component of membrane.” It is noteworthy that most of the genes associated with the term “integral component of membrane” are in fact transporters, namely, MFS transporter, ABC-2 type transporter and ammonium/hexose transporters. MFS transporters are the most common category of secondary carrier proteins. Members of this group are involved in the uptake of essential minerals and nutrients, also functioning as nutrient sensors, while others are responsible for the transport of various drugs and toxins (Liu et al., 2017). Moreover, MFS transporters also play an important role in cellular resistance to oxidative stress in *Alternaria alternata* (Chen et al., 2017) and in some cases can act as virulence factors (Liu et al., 2017). In turn, ABC transporters confer tolerance by efflux of compounds across the membrane, thereby preventing an increase in intracellular concentration of toxic substances (Coleman et al., 2011). Hexose transporter, on the other hand, is vital for the fungi to access the organic carbon sources of their host (Wahl et al., 2010). Finally, the ammonium secretion carried out by its transporter in developing hyphae contributes to the necrotrophic colonization of *Colletotrichum* spp. (Miyara et al., 2012; O’Connell et al., 2012; Shnaiderman et al., 2013) but, at early stages of spore germination, ammonia modulates the induction of appressorium formation (Miyara et al., 2010). Other genes found in the “integral component of membrane” category, code for instance for a tetraspanins which have been also implicated in fungi appressorium mediated penetration in *Botrytis cinerea* (Gourgues et al., 2004). Genes encoding cytochrome P450s are highly represented in both enriched categories (“oxidation–reduction process” and “integral component of membrane”) and this protein has an important role in primary and secondary metabolism, and fungal pathogenicity (Rao and Nandineni, 2017). Additionally, the most represented genes falling in the “oxidation–reduction process” category encode proteins closely involved in the detoxification of drugs produced by the plant in an effort to shield itself from the invader, such as FAD dependent oxidoreductase, Short chan dehydrogenase (SDS) and nitric oxide synthase (NOS) (Wang and Higgins, 2005; Kwon et al., 2010; Sygmund et al., 2011). The mutant phenotype of SDS and NOS in *Magnaporthe oryzae* and *Colletotrichum coccodes*, respectively, showed modifications in the pathogen efficiency to penetrate the host in the initial phase of the infection process (conidia production and germination) (Wang and Higgins, 2005; Kwon et al., 2010). Within this functional category, a gene coding for a taurine catabolism dioxygenase (TauD) was also found. This enzyme is involved in taurine degradation, catalyzing it into sulfite and aminoactyledhyde, and under starvation conditions, taurine can be used as a sulfur source by some microorganisms (Yew et al., 2016). Therefore, most of the genes representative of these two enriched categories are involved in biological processes able to give a survival advantage of the pathogen in an adverse environment, like host colonization.

The analysis of the dN/dS ratio on the *filtered_dataset* showed that 258 genes could be under positive selection (*ps_filtered_dataset*), from which 26 have more than one non-synonymous mutation. The potential role of these genes in fungal pathogenicity and virulence was assessed by a BLAST search against the PHI-base. A total of 30% (77 genes) had homology, and 15% (40 genes) were described as important for fungal pathogenicity and virulence when a mutant phenotype was produced. Five of them were identified as genes required for pathogenicity in other fungi, inducing mutant phenotypes of “total loss of pathogenicity” (encoding chitin synthase, GTP-binding protein, ABC transporter, alpha-mannosyltransferase cmt1 and cytochrome p450) and the remaining 36 genes, including the ones responsible for a change in virulence, are mainly involved in oxidative responses (for instance, cytochrome P450) and transport (mainly, ABC Superfamily and MFS transporters). Once again, these two biological processes stand out in our study and their importance is well-documented in the literature (Buiate et al., 2017; Chen et al., 2017; Liu et al., 2017; Rao and Nandineni, 2017; Zeng et al., 2018). A comparative genomic analysis between two *Colletotrichum* species (*C. sublineola* and *C. graminicola*) in different hosts, showed an enrichment of proteins of these classes in the non-conserved proteins dataset, with transporters being the most represented PFAM category (Buiate et al., 2017). Additionally, the enrichment of genes encoding proteins related to oxidative responses can be a result of host–pathogen evolution, since reactive oxygen species (ROS) have been described as vital for stress responses, programmed cell death and plant defenses (Silva et al., 2006). Finally, transcription factors (TFs) were also highly represented, especially “the fungal specific transcription factor” and “C6 transcription factor,” which could suggest that changes in gene expression patterns may be also important for *C. kahawae’s* pathogenicity. In conclusion, most of the loci with non-synonymous mutations and genes of under positive selection with an enriched representation were associated with transporters, oxidative response, and signaling, suggesting an important role for these biological processes in the adaptation of *C. kahawae* to *C. arabica*, and providing candidate genes for evolutionary changes. Similar findings were also reported by Buiate et al. (2017) and Rao and Nandineni (2017) based on different genomic comparative analyses within the genus *Colletotrichum*, which suggest that these adaptive mechanisms could be globally associated to varying aspects of each host environment, and to the secretion of or evasion to toxic secondary metabolites. In this sense, host specificity in closely related pathosystems of the *Colletotrichum* genus could be not only a matter of pathogen recognition, but also a much broader adaptation to the living host environment across the entire course of pathogen development, which has presumably occurred during co-evolution of the host and its pathogen.

Nevertheless, it is important to note that only around ∼30% of the RAD loci contained in each dataset was mapped or annotated due to the lack of a properly annotated reference genome. Associated with this, all the genomic variation located in non-coding regions was not further studied, and consequently, a high amount of information may have not been retrieved. Moreover, the reproductive characteristics and the evolutionary history of *C. kahawae*, make it difficult to separate the demographic signal from the selection pattern. In fact, it has been proposed that *C. kahawae* is a true clonal pathogen that has emerged by a host-jump from a non-pathogenic group (Silva et al., 2012). In such scenario, *C. kahawae* was subjected to a strong disruptive selection during the first stages of the adaptation to *C. arabica*. According to Grünwald et al. (2016), asexual reproduction could greatly amplify new advantageous mutations to extremely high frequencies along the entire genome by hitchhiking, and not just at the neighboring genes. This would eliminate polymorphisms and maintain only the intact genome of those individuals in the population that had the favored mutations, resulting in a strong genetic bottleneck and the lack of shared polymorphisms with the remaining non-pathogenic fungi. Thus, in a perfectly clonal pathogen, each adaptive allele that arises, will be linked to every other allele in the genome, and consequently the selection is more likely to act at the level of individual clones than individual alleles (Shapiro et al., 2009; Grünwald et al., 2016; Plissonneau et al., 2017). In this sense, if the goal is to distinguish adaptive loci from other fixed mutations in a clonal background, the typical genome-scan may be a limited approach, and hence it is crucial to look for the excess of functional changes, such as the enrichment of non-synonymous mutations and search for genes putatively under selection (Shapiro et al., 2009; Plissonneau et al., 2017).

### Genome-Wide Association Study of Aggressiveness in *C. kahawa*e

Based on the phenotypic evaluation of 26 *C. kahawae* isolates performed by Vieira et al. (2018), a genome-wide association analysis was conducted in order to better understand the genetic mechanisms underlying aggressiveness. According to Dalman et al. (2013), applying a GWAS to an organism in a haploid stage, which can be clonally reproduced in high numbers and phenotyped repeatedly, increases the accuracy of the phenotypic measurements and the power of the association analysis in several orders of magnitude, when compared to diploids. For this reason, a low number of haploid individuals can be used to successfully identify robust associations in small fungal genomes, whereas several 100s of individuals are needed in diploid organisms with large genomes such as humans and plants (Dalman et al., 2013).

In *C. kahawae*, the number of SNPs that are not associated with population structure is low (173 SNPs) and correspond to only 5% of the total genetic variation. No causal SNPs were identified in this dataset, but instead a group of SNPs of small effect was detected. In single-SNP association analyses, the 10 individual SNPs found to be associated (BF0.97) showed a low phenotypic effect in aggressiveness (β| = 0.059 to |β| = 0.087) for all the pairwise analyses (*High* vs. *Moderate*, *High* vs. *Low*, *Low* vs. *Moderate*), but in the continuous analysis (*AUDPC*) 5 SNPs presented a moderate phenotypic effect (β| = 1.24). Despite these differences, three of the SNPs associated with aggressiveness in the continuous analysis were also found in the pairwise analyses.

In the multi-SNP association test, 15 SNPs with a posterior inclusion probability of 97.5% (PIP0.975) were found, showing moderate effect for all pairwise analyses (*High* vs. *Moderate*, *High* vs. *Low*, *Low* vs. *Moderate*) [PIP0.97 SNPs |β| > 0,36] and for the continuous analysis (*AUDPC*) [PIP0.97 SNPs |β| > 0,62]. The phenotypic effect seems to be smaller in all pairwise analyses that included the *High* aggressiveness class, probably due to the low number of isolates that compose this class. Moreover, 7 SNPs were common to the SNPs identified in single SNP analyses, specifically 1 in 5 SNPs was common to both approaches in *High* vs. *Low* and *High* vs. *Moderate* pairwise analyses, 3 in 4 for *Low* vs. *Moderate* pairwise analysis, and 3 in 5 for the continuous analysis with *AUDPC*. These results provide additional support for the reliability of the *Low* vs. *Moderate* and *AUDPC* analyses.

The annotation of the loci containing the total of SNPs putatively associated with aggressiveness allowed the identification of three candidate genes in the single-association analysis (F-box domain-containing, nitrosoguanidine resistance and Fungal specific transcription factor domain-containing), and two candidate genes in the multi-SNP association analysis (nitrosoguanidine resistance and C6 transcription factor). The gene coding for a nitrosoguanidine resistance protein, which is an integral component of the membrane that is able to regulate the fungal-type cell wall organization and phospholipid translocation (García-López et al., 2010; García-Marqués et al., 2016), was the only candidate common to both approaches. In addition, among the few candidate genes identified, two encode transcription factors located in the nucleus, which may suggest that differential gene expression and/or associated regulatory mechanisms might have a preponderant role in aggressiveness. However, none of these genes was previously associated with aggressiveness in other plant–pathogen interactions, which suggests that they may be specific of the *C. arabica – C. kahawae* interaction. Some of these SNPs were also found in genes annotated as hypothetical proteins, in which for two of them, orthologs to the *GIT3* and *srbA* genes of *Candida albicans* and *Aspergillus fumigatus*, respectively, the mutant phenotypes showed a reduced virulence. SrbA belongs to the basic Helix-Loop-Helix (bHLH) family of transcription factors and is crucial for antifungal drug resistance, hypoxia adaptation, and virulence (Chung et al., 2014), while glycerophosphoinositol permease GIT3 is required for early post-inoculation growth, transport activity and full virulence (Bishop et al., 2013). The remaining significantly associated SNPs were in intergenic regions, or in not annotated loci. These SNPs may represent regulatory elements or unknown genes that are responsible for the trait variation.

Finally, the success and power of an association study relies on the number of SNP markers and on the LD decay. In *C. kahawae*, both conditions are far from ideal, as a low number of SNPs not related with the population structure pattern was identified and the entire genome is inherited asexually as a single, non-recombining linkage group, which can increase the number of false positives. Therefore, as in any GWA study, a further detailed investigation of these candidate genes is required to confirm their involvement in *C. kahawae’s* aggressiveness and assess their causative effect at the phenotypic level.

## Conclusion

This work took the first step toward the understanding of the genetic mechanisms underlying the ability of *C. kahawae* to infect green coffee berries and its aggressiveness. Our results suggest that *C. kahawae’s* pathogenicity involves several biological processes such as detoxification and transport, regulation of host and pathogen gene expression, and signaling. Fifteen percent of the genes under selection were described as having an important role in fungal pathogenicity and virulence, being some of them identified as genes proven to lead to the total loss of pathogenicity in other fungi when mutated. Finally, the high abundance of TFs may suggest that expression changes in gene expression patterns can be more important than the presence/absence of individual gene alleles. On the other hand, aggressiveness does not seem to be regulated by any causal mutation and even the associated SNPs are of small effect, which leads to three possible conclusions: (i) aggressiveness is regulated by a set of many small effect SNPs difficult to detect with a GWAS analysis; (ii) aggressiveness is a plastic trait regulated by differential gene expression and associated regulatory mechanisms, consequently, a transcriptome and epigenome analysis is needed to complement the current study; (iii) aggressiveness is not under selection and is governed by physiological conditions. Nevertheless, a repertoire of candidate genes is now provided that can be studied through gene expression and additional functional analyses (knockouts, knockdowns and transgenics) to ascertain their causative role in *C. kahawae* aggressiveness and pathogenicity. Finally, the collected information may be of use to the development of future evidence-based sustainable control measures.

## Author Contributions

AV, VV, OP, and DB designed the experiments. AV performed the experiments and wrote the manuscript. AV and DS analyzed the experimental data. VV, OP, and DB contributed reagents, materials, and analysis tools. All authors listed have made a substantial, direct and intellectual contribution to the work, and approved it for publication.

## Conflict of Interest Statement

The authors declare that the research was conducted in the absence of any commercial or financial relationships that could be construed as a potential conflict of interest.

## Footnotes

1 https://github.com/OdiogoSilva/TriFusion

2 http://tree.bio.ed.ac.uk/software/figtree/

3 https://inkscape.org/pt/

4 useast.ensembl.org/index.html
