# Supplementary figures and images for: Genome-Wide Signatures of Selection in Colletotrichum kahawae Reveal Candidate Genes Potentially Involved in Pathogenicity and Aggressiveness
Source: Front Microbiol. 2019 Jun 19;10:1374. doi: 10.3389/fmicb.2019.01374 (PMC6593080; doi:10.3389/fmicb.2019.01374)

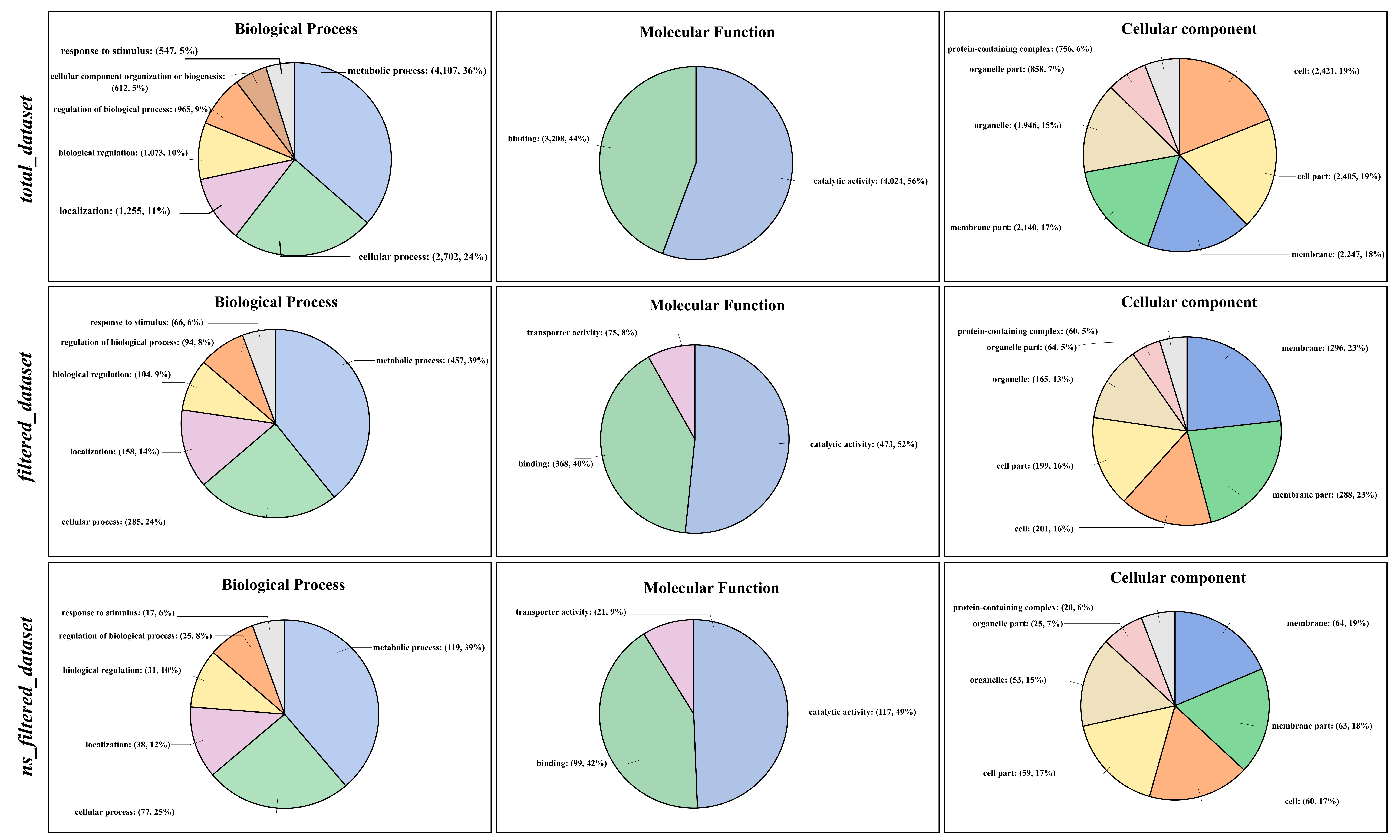

Supplement: Supplementary file 1 [file Image_1.TIF]

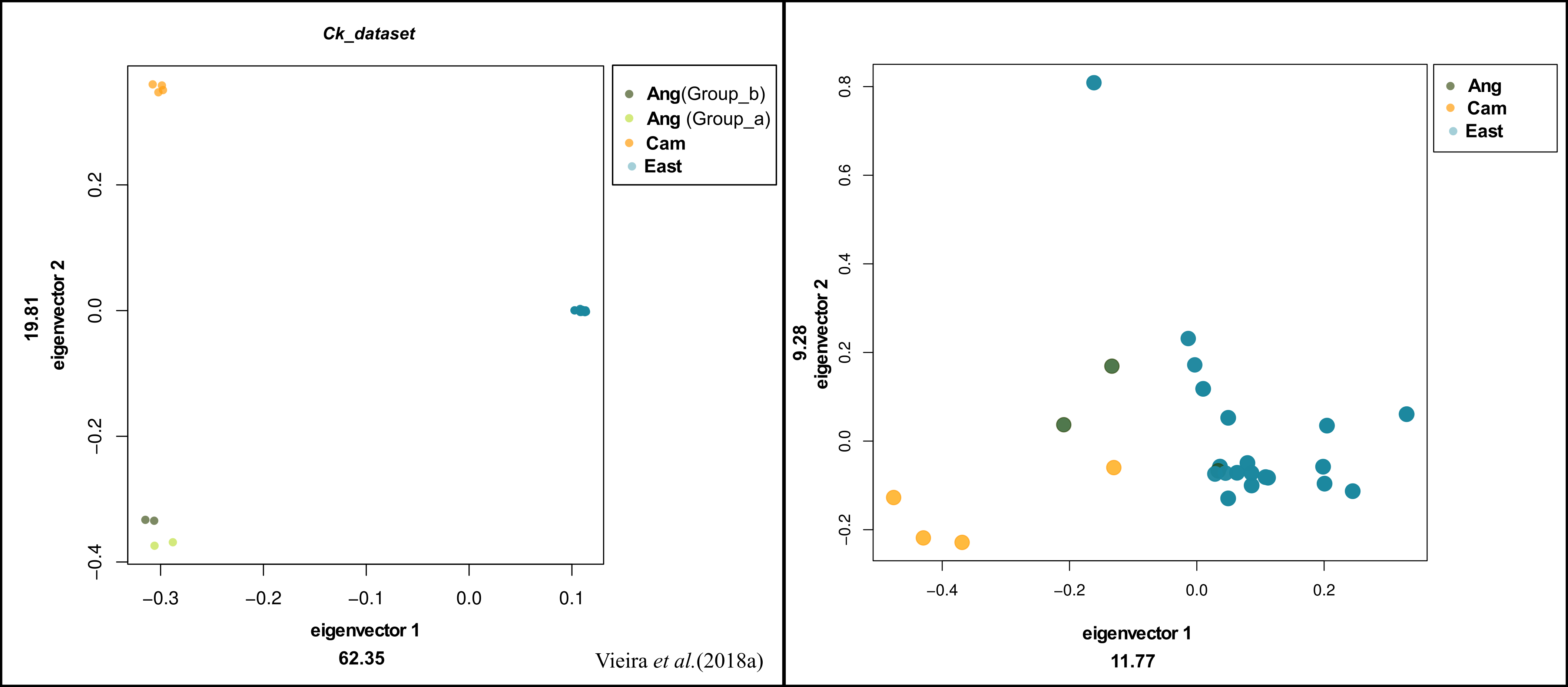

Supplement: Supplementary file 2 [file Image_2.TIF]

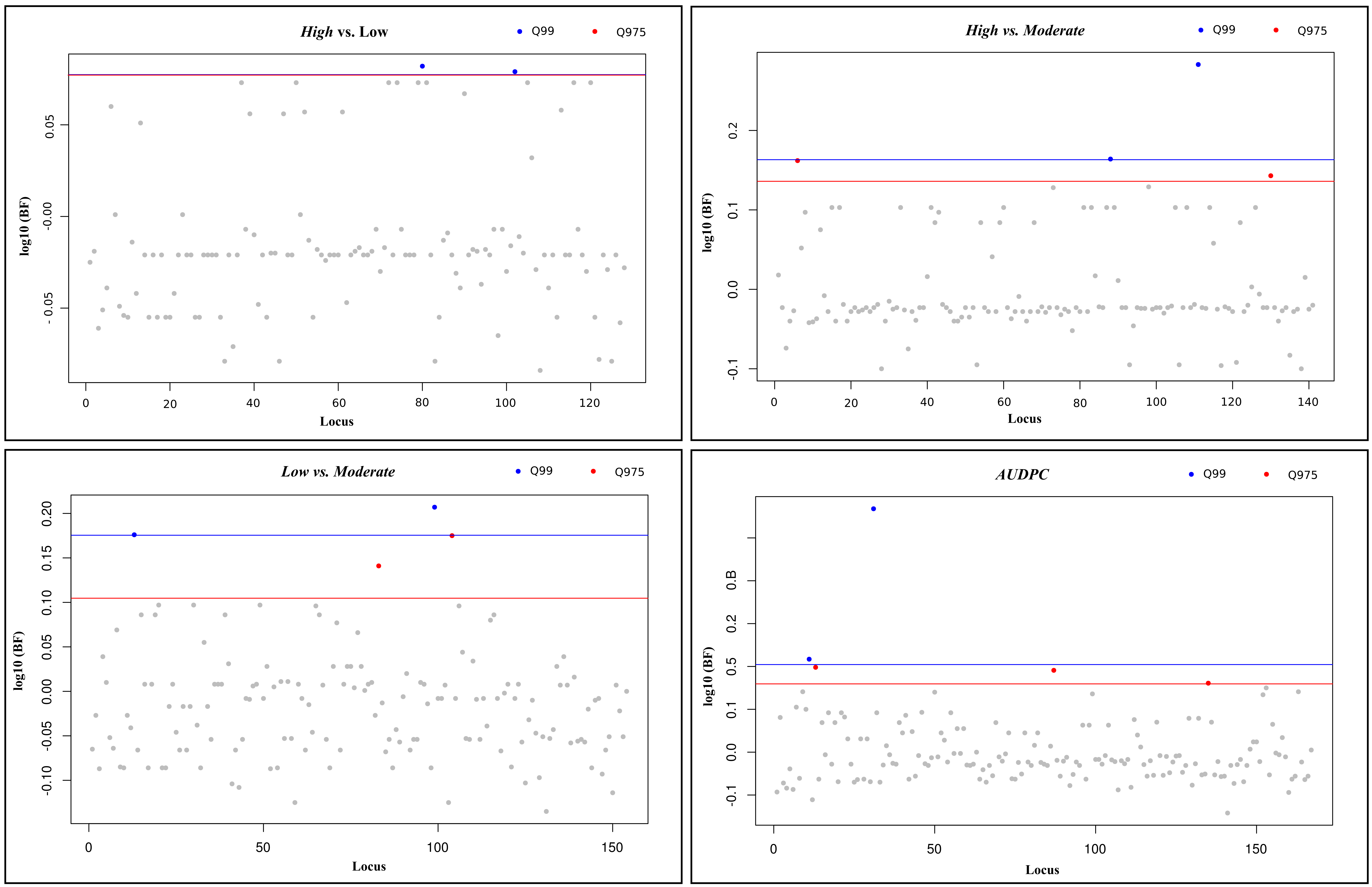

Supplement: Supplementary file 3 [file Image_3.TIF]
